# Suppression of telomere capping defects of *Saccharomyces cerevisiae yku70* and *yku80* mutants by telomerase

**DOI:** 10.1093/g3journal/jkab359

**Published:** 2021-10-13

**Authors:** Cory L Holland, Brian A Sanderson, James K Titus, Monica F Weis, Angelica M Riojas, Eric Malczewskyj, Brian M Wasko, L Kevin Lewis

**Affiliations:** 1 Department of Chemistry and Biochemistry, Texas State University, San Marcos, TX 78666, USA; 2 Department of Biology and Biotechnology, University of Houston-Clear Lake, Houston, TX, 77058, USA

**Keywords:** telomerase, end-joining, recombination, replication, checkpoint, DNA repair, senescence

## Abstract

The Ku complex performs multiple functions inside eukaryotic cells, including protection of chromosomal DNA ends from degradation and fusion events, recruitment of telomerase, and repair of double-strand breaks (DSBs). Inactivation of Ku complex genes *YKU70* or *YKU80* in cells of the yeast *Saccharomyces cerevisiae* gives rise to mutants that exhibit shortened telomeres and temperature-sensitive growth. In this study, we have investigated the mechanism by which overexpression of telomerase suppresses the temperature sensitivity of *yku* mutants. Viability of *yku* cells was restored by overexpression of the Est2 reverse transcriptase and *TLC1* RNA template subunits of telomerase, but not the Est1 or Est3 proteins. Overexpression of other telomerase- and telomere-associated proteins (Cdc13, Stn1, Ten1, Rif1, Rif2, Sir3, and Sir4) did not suppress the growth defects of *yku70* cells. Mechanistic features of suppression were assessed using several *TLC1* RNA deletion derivatives and Est2 enzyme mutants. Supraphysiological levels of three catalytically inactive reverse transcriptase mutants (Est2-D530A, Est2-D670A, and Est2-D671A) suppressed the loss of viability as efficiently as the wild-type Est2 protein, without inducing cell senescence. Roles of proteins regulating telomere length were also determined. The results support a model in which chromosomes in *yku* mutants are stabilized via a replication-independent mechanism involving structural reinforcement of protective telomere cap structures.

## Introduction

The evolutionarily conserved Ku complex consists of two proteins, Ku70 and Ku80 (also called Ku86), that play critical roles in stabilization of chromosomes and in maintenance of genome integrity. The complex is required in humans and animals for the generation of antibody diversity and immunocompetence. In addition, because the complex is essential for DNA repair and acts to protect telomeres, it also affects diverse processes such as aging and cancer (Fisher and Zakian 2005; Gullo *et al.* 2006; Fell and Schild-Poulter 2015). The two Ku proteins associate with each other to form a ring-shaped, dimeric complex that has high affinity for the ends of linear double-stranded DNA. This affinity is utilized in at least two major ways inside cells: for rejoining of DNA double-strand breaks (DSBs) and for stabilization and replication of chromosome ends (Downs and Jackson 2004; Fisher and Zakian 2005; Gullo *et al.* 2006; Grundy *et al.* 2014; Fell and Schild-Poulter 2015; Westmoreland *et al.* 2018).

DNA DSBs are generated inside cells through both internal processes and via exposure to external DNA damaging agents. These types of lesions can be repaired by either of two pathways, called nonhomologous end-joining (NHEJ) and homology-directed repair (HDR). The latter pathway is also often referred to as homologous recombination (HR). Both pathways are utilized in eukaryotic cells, but organisms typically use one more frequently than the other. NHEJ is the dominant pathway in human cells, but HDR is generally preferred in cells of the budding yeast *Saccharomyces cerevisiae*. Repair of DSBs by NHEJ involves the sequential actions of many proteins, and is initiated by binding of the Ku complex (Lewis and Resnick 2000; Dudásová *et al.* 2004; Daley *et al.* 2005; Hefferin and Tomkinson 2005; Mahaney *et al.* 2009; Lieber 2010a; Davis and Chen 2013). After association with DSB ends, Ku performs two major functions: (1) protection of the ends from degradation or modification by nucleases and other enzymes and (2) recruitment and stabilization of multiple proteins including the DNA ligase IV complex (Grob *et al.* 2012), whose activity is ultimately responsible for the formation of new covalent bonds between individual DNA strands. Ku plays an important role in regulating pathway choice (NHEJ *vs* HR), especially in promoting NHEJ repair during G_1_ phase in wild-type cells and in suppressing recombinational repair in certain yeast strain backgrounds (Zhang *et al.* 2007; Clerici *et al.* 2008; Wasko *et al.* 2009; Shim *et al.* 2010; Daley and Sung 2014; Mathiasen and Lisby 2014). During NHEJ repair, Ku may also be involved in processing of lesions near the broken ends via an intrinsic dRP/AP lyase activity (Roberts *et al.* 2010; Strande *et al.* 2012). Other enzymatic functions that have been ascribed to the Ku complex, but which have not yet been firmly established, include deubiquitinase, DNA helicase, and ATPase activities (Lewis and Resnick 2000; Dudásová *et al.* 2004; Rathaus *et al.* 2009; Lieber 2010b).

In addition to its role in DNA repair, Ku also performs several important functions at chromosome ends, where it associates with telomeric DNA. In *S. cerevisiae* cells, Ku protects the ends from undesirable events such as nuclease degradation, recombination, and formation of end-to-end fusions. It also plays important roles in recruitment of the telomerase complex, silencing of transcription, and tethering of chromosome ends to nuclear membrane pore complexes (Gravel *et al.* 1998; Fisher and Zakian 2005; Taddei and Gasser 2012; Nandakumar and Cech 2013; Avşaroğlu *et al.* 2014; Williams *et al.* 2014).

Yeast mutants with either of the two Ku genes inactivated (*YKU70* or *YKU80*) exhibit a number of phenotypes. The cells are defective in assays that specifically measure the efficiency of DSB repair by NHEJ, exhibit modest strain background-dependent sensitivities to DNA damaging agents, and have altered rates of some types of chromosomal DNA recombination (Barnes and Rio 1997; Cervelli and Galli 2000; Lewis and Resnick 2000; Fasullo *et al.* 2005; Hefferin and Tomkinson 2005; Yamana *et al.* 2005; Letavayová *et al.* 2006). When grown at normal temperatures (at or below 30°C), the mutant cells contain short but stable telomeres that have long single-stranded DNA tails throughout the cell cycle (Fisher and Zakian 2005; Fell and Schild-Poulter 2015). The mutants also exhibit reductions in transcriptional silencing (telomere position effect) and telomere tethering at the nuclear periphery (Downs and Jackson 2004; Fisher and Zakian 2005; Taddei and Gasser 2012).

When shifted to 37°C, *yku70* and *yku80* null mutants undergo a DNA damage-induced cell cycle checkpoint response, arrest growth in G_2_ phase, and lose viability. At the elevated temperature, protein cap structures break down and the telomeres shorten progressively at the rate of about 10–15 bp per generation (Gravel and Wellinger 2002). The cell cycle arrest is dependent upon a subset of known DNA damage checkpoint genes, including *RAD9*, *RAD24*, *RAD53*, *MEC1*, and *CHK1* (Teo and Jackson 2001; Maringele and Lydall 2002). Telomeric single-stranded DNA is increased at the higher temperatures, and this may serve as the checkpoint arrest signal (Maringele and Lydall 2002; Fisher and Zakian 2005). The increased single-stranded DNA, checkpoint response, and death of *yku* cells at elevated temperatures can be largely suppressed by deletion of *EXO1*, which encodes a 5′-to-3′ exonuclease. Evidence suggests that Exo1 degrades chromosome ends whenever telomere cap structures are compromised (Maringele and Lydall 2002, 2004; Vega *et al.* 2007; Bonetti *et al.* 2010; Dewar and Lydall 2010). Growth of *yku* mutants at 37°C is also rescued, at least partially, when other genes such as the telomerase regulator *RIF1* and a small number of other genes associated with DNA damage-responsive and mitotic spindle checkpoints (*RAD9*, *CHK1*, *MEC1*, *CGI121*, and *MAD2*) are inactivated (Gravel and Wellinger 2002; Maringele and Lydall 2002; Downey *et al.* 2006). Furthermore, a screen by Addinall *et al.* (2011) identified several additional genes that affect the growth of *yku70* cells at elevated temperatures, either positively or negatively, when they are inactivated.

The *S. cerevisiae* core telomerase complex consists of three proteins (Est1, Est2, and Est3) plus an RNA subunit encoded by the *TLC1* gene. Est2 is the catalytic subunit, exhibiting RNA-dependent DNA polymerase activity, and *TLC1* RNA serves as the template for new DNA synthesis (Chan and Blackburn 2004; Wellinger and Zakian 2012; Teixeira 2013). Overexpression of either Est2 protein or *TLC1* RNA can suppress the inviability of *yku* mutants at 37°C (Nugent *et al.* 1998; Teo and Jackson 2001; Lewis *et al.* 2002; Tong *et al.* 2011). The effectiveness of overexpression of the Est1 subunit was variable in two previous studies (Nugent *et al.* 1998; Tong *et al.* 2011). Another gene, *RTT103*, implicated in DNA repair and transcription termination but not telomerase function, has been found to partially suppress killing of *yku70* cells at 37°C when overexpressed (Srividya *et al.* 2012).

The mechanism by which supraphysiological levels of Est2 reverse transcriptase can stabilize telomeres and rescue the inviability of *yku70* and *yku80* mutants at elevated temperatures is unknown. In the current study, we have investigated the roles of several genes affecting telomere metabolism in this process, including genes involved in DNA repair, replication, chromatin structure, and tethering of telomeres to nuclear porins. The results suggest that elevated Est2 protein stabilizes telomeres via a reverse transcriptase activity-independent mechanism involving formation of telomere-associated protein complexes with enhanced resistance to degradation by Exo1 nuclease.

## Materials and methods

### Bacteriological and yeast media and preparative methods

Nonselective YPDA and YPG yeast growth media and synthetic media with drop-out mix were prepared as described (Sherman 2002). For selection of resistant strains, G418 sulfate and hygromycin B were added to media at concentrations of 200 and 250 µg/ml, respectively. *Escherichia coli* cells were grown in LB or TB broth-based media (Green and Sambrook 2012). The high-efficiency lithium acetate method described by Gietz and Schiestl was used for DNA transformations involving gene disruption (Gietz and Schiestl 2007) and a rapid lithium acetate/DMSO transformation method was used for simple plasmid transformations (Tripp *et al.* 2013). Yeast chromosomal DNA was purified as described (Lee *et al.* 2012). Plasmid DNAs were purified using Qiagen Qiaprep kits. PCR reactions utilized an Applied Biosystems 2720 Thermal Cycler. Restriction enzymes, Antarctic phosphatase, RNase I_f__,_ and Phusion high-fidelity DNA polymerase were obtained from New England Biolabs. The chemical 5-fluoroorotic acid (5-FOA) was purchased from Gold Biotechnology.

### Yeast strains and plasmids

Most experiments employed haploid yeast strains derived from BY4742 (*MATα his3*Δ*1 leu2Δ0 lys2Δ0 ura3Δ0*) (Brachmann *et al.* 1998). A list of all strains constructed for the study is presented in Table  1. Gene inactivations were accomplished by performing PCR fragment-mediated gene disruption or by using previously constructed gene disruption-deletion plasmids. Plasmids used in the study included the vectors pRS316, pRS425, pRS426, and p426TEF (Christianson *et al.* 1992; Mumberg *et al.* 1995), pFA6MX4 (G418^r^) and pAG32 (Hygromycin B^r^) (Goldstein and McCusker 1999), gene disruption-deletion plasmids p52Blast *(rad52Δ::hisG-URA3-hisG*) and p*Δ*52Leu (*rad52Δ::LEU2*) (Lewis *et al.* 1998), pLKL64Y (*2µ LEU2 ADH1p::TLC1*), pLKL82Y (*CEN/ARS URA3 GAL1-V10p::EST2*) and pLKL83Y (*2µ URA3 ADH1p::TLC1*) (this work and Becerra *et al.* 2012), pVL715 (*2µ URA3 ADH1p::EST2*), pVL799 (*2µ LEU2 ADH1p::TLC1*) and pVL999 (*2µ LEU2 ADH1p::EST2*) (Nugent *et al.* 1998), pGEM4Z S-H/URA (Porter *et al.* 1996), and pCDNA50.1 (*CEN/ARS URA3 GAL1p::EXO1*) (Lewis *et al.* 1998). Construction of plasmids used for expression of TLC1 RNA deletion derivatives (pLKL74Y—pLKL79Y, pTRP61, and pTCG-3xStem) was described previously (Stellwagen *et al.* 2003; Wasko *et al.* 2009). Plasmids YEp195-TEN1-HA-HIS and YEp195-STN1-HA-HIS (Grandin *et al.* 2001), pVL459 (*2µ, URA3 CDC13*) (Chandra *et al.* 2001), pRF4-6NL (*2µ TRP1 GAL1p*), pRF4-6NL+RIF1 (*2µ TRP1 GAL1p::RIF1*), pRF4-6NL+RIF2 (*2µ TRP1 GAL1p::RIF2*), pRF4-6NL+SIR3 (*2µ TRP1 GAL1p::SIR3*), pRF4-6NL+SIR4 (*2µ TRP1 GAL1p::SIR4*) (Bourns *et al.* 1998) have been described.

**Table 1:** Yeast strains employed in the study

Strain	Genotype	Source
BY4742	*MATα his3Δ1 leu2Δ0 lys2Δ0 ura3Δ0*	Brachmann *et al.* (1998)
VL6α	*MATα ura3-52 trp1(Δ63) lys2-801 his3-Δ200 met14 ade2-101*	Lewis *et al.* (1998)
YLKL494	VL6α, *yku70Δ::TRP1*	This work
YLKL502	VL6α, *yku70Δ::TRP1 rad52Δ::hisG*	This work
YLKL611	VL6α, *yku70Δ::TRP1 dnl4Δ::G418^r^*	This work
YLKL652	BY4742, *yku70Δ::HIS3*	This work
YLKL814	BY4742, *yku70Δ::HIS3 trp1Δ::hisG-URA3-hisG*	This work
YLKL868	BY4742, *sir2Δ*::*HygB^r^ yku70Δ*::*URA3*	This work
YLKL869	BY4742, *rif1Δ*::*HygB^r^ yku70Δ*::*URA3*	This work
YLKL870	BY4742, *rif2Δ*::*HygB^r^ yku70Δ*::*URA3*	This work
YLKL871	BY4742, *sir4Δ*::*G418^r^ yku70Δ*::*URA3*	This work
YLKL956	BY4742, *yku80Δ::G418^r^ yku70Δ*::*URA3*	This work
YLKL982	BY4742, *exo1Δ*::*G418^r^ yku70Δ*::*URA3*	This work
YLKL983	BY4742, *mms4Δ*::*G418^r^ yku70Δ*::*URA3*	This work
YLKL984	BY4742, *rad1Δ*::*G418^r^ yku70Δ*::*URA3*	This work
YLKL985	BY4742, *rad10Δ*::*G418^r^ yku70Δ*::*URA3*	This work
YLKL986	BY4742, *mus81Δ*::*G418^r^ yku70Δ*::*URA3*	This work
YLKL991	BY4742, *yku70Δ::HIS3 tlc1Δ::HygB^r^* + pLKL83Y (*URA3 ADH1p::TLC1*)	This work
YLKL992	BY4742, *sir3Δ*::*G418^r^ yku70Δ*::*URA3*	This work
YLKL1184	BY4742, *sae2Δ*::*G418^r^ yku70Δ*::*URA3*	This work
YLKL1185	BY4742, *srs2Δ*::*G418^r^ yku70Δ*::*URA3*	This work
YLKL1697	BY4742, *est1Δ::G418^r^* + pLKL92Y (*2µ URA3 TEF1p::EST1*)	This work
YLKL1698	BY4742, *est3Δ::G418^r^* + pLKL93Y (*2µ URA3 TEF1p::EST3*)	This work

The plasmid pLKL92Y (*2µ URA3 TEF1p::EST1*) was created by cutting p426TEF with SpeI and XhoI, treating with Antarctic phosphatase, and ligating to SpeI+XhoI-cut *EST1* gene PCR DNA. *EST1* was amplified from BY4742 chromosomal DNA using Phusion DNA polymerase along with primers Est1A (GACTCAAACTAGTAAGCTTGATAATGGATAATGAAG) and Est1B (GACTCAACTCGAGAAG CTTTACTTGTTCTCTCAAGT). Plasmid pLKL93Y (*2µ URA3 TEF1p::EST3*) was constructed by digesting p426TEF with SpeI, treating with phosphatase, and ligating to SpeI-cut *EST3* gene PCR fragments. *EST3* was amplified using primers Est3A (GACTCAAACTAGTAAGCTTGTAAACAATGCCGAAAG) and Est3B (GACTACAACTAGTAAGCTTGTTTCTCTAGAGGAGTA). Proper orientation was confirmed prior to sequencing by cutting with both SacI and MscI. Plasmid promoters and inserts were sequenced on both strands (Retrogen, Inc.). The senescence phenotype of *est1* cells observed using streak plate assays (Becerra *et al.* 2012) was suppressed by transfer of pLKL92Y (*TEF1p::EST1*) into the cells. Similarly, plasmid pLKL93Y (*TEF1p::EST3*) abolished the senescence phenotyope of *est3* mutants. Strains created for these experiments included YLKL1697 (BY4742, *est1Δ::G418^r^* + pLKL92Y) and YLKL1698 (BY4742, *est3Δ::G418^r^* + pLKL93Y). The *EST1* and *EST3* genes in these strains were knocked out after first inserting the appropriate plasmid into the cells; senescence could be monitored after loss of the plasmid was subsequently selected for using 5-FOA plates.

Yeast strains YLKL868, YLKL869, YLKL870, YLKL871, YLKL982, YLKL983, YLKL984, YLKL985, YLKL986, YLKL956, and YLKL992 (Table  1) were constructed by transforming single mutant cells obtained from a BY4742 *MATα* library (Open BioSystems) (McKinney *et al.* 2013) with HindIII/EcoRI-digested *YKU70* deletion plasmid pGEM4Z S-H/URA. Cells from Ura^+^ colonies were streaked to synthetic glucose plates lacking uracil and grown at 30°C. Chromosomal DNAs from the resulting colonies were then analyzed by PCR with 5'Ku70 and 3'Ku70 test primers to identify transformants with proper insertions. Primer sequences are available upon request.

YLKL991 was created from YLKL652 (*yku70Δ*::*HIS3*). YLKL652 cells containing pLKL83Y (*ADH1*p::*TLC1 URA3*) were employed initially: the Hygromycin B resistance gene on pAG32 was PCR amplified using 5’gtlc1AA and 3’ gtlc1BB primers and the resulting *tlc1Δ::HygB^r^* fragment was transformed into the cells. Colonies containing cells with *tlc1Δ::HygB^r^* disruptions were confirmed by PCR using 5′TLC1 and 3′TLC1 test primers.

Strains used to test requirements for *RAD52* and *DNL4* were VL6α, YLKL494 YLKL502, and YLKL611 (Table  1). Plasmids pRS316 and pVL715 (*2µ URA3 ADH1p::EST2*) were used for experiments with VL6α-derived strains. Expression of catalytically inactive Est2 proteins was accomplished using plasmids pVL735 (*ADH1p::est2-D530A*), pVL743 (*ADH1p::est2-D670A*), and pVL744 (*ADH1p::est2-D671A*). The *EST2* genes within pVL715, pVL735, pVL743, and pVL744 were resequenced for this project and changes at codons 530 (GAT to GCT), 670 (GAC to GCC), and 671 (GAT to GCT) were confirmed in the latter three plasmids. Strain YLKL814 (BY4742, *yku70Δ::HIS3 trp1Δ::hisG-URA3-hisG*) was employed for overexpression of *RIF1*, *RIF2*, *SIR3*, and *SIR4* from *TRP1*-containing plasmids.

### Analysis of survival of telomerase-deficient *tlc1* strains and cellular senescence

Tests performed to determine if *TLC1* RNA was required for rescue of *yku70* cells by overexpression of Est2 used YLKL652 (BY4742, *yku70Δ::HIS3*) and YLKL991 [BY4742, *yku70Δ::HIS3 tlc1Δ::HygB^r^* + pLKL83Y (*URA3 ADH1p::TLC1*)]. YLKL991 cells were transformed with pRS425 (*LEU2*) and pVL999 (*LEU2 ADH1p::EST2*) prior to selection for cells that had lost the *TLC1* plasmid on synthetic plates containing 0.1% 5-FOA without leucine (Boeke *et al.* 1987). The control strain used to monitor telomere-initiated cell senescence was YLKL803, a BY4742-derived *est2* mutant containing the plasmid pLKL82Y (*GAL1-V10p::EST2*, *URA3*, and *CEN/ARS*) (Becerra *et al.* 2012). This strain was propagated on synthetic galactose plates without uracil and then streaked to glucose complete plates to initiate and monitor senescence. Repetitive picking and streaking of individual colonies from one plate to another result in senescence (loss of growth capability) that is visible on the 4th streak if medium-sized colonies are picked each time and on the 5th streak plate when small colonies are picked (Becerra *et al.* 2012; Ghanem *et al.* 2019). Counting cells within the small colonies used in this study indicated that each had undergone approximately 18–19 generations before being picked and streaked to a new plate. In *est2* cells senescence was observed after approximately 70 generations of growth.

### Southern blot and cell cycle phase analysis experiments with catalytically inactive *est2* mutants

For cell cycle phase analysis experiments, BY4742 cells and *yku70* cells containing pRS426, pVL715, pVL735, or pVL743 (vector, *EST2*, *est2-D530A*, and *est2-D670A* plasmids, respectively) were inoculated from a fresh glucose minus uracil plate into YPDA broth at 3 × 10^6^ cells per ml and shaken at 37°C for 12 h. Telomere degradation-induced large-budded (G_2_/M) cells were scored as cells with buds that were ≥50% of the size of the mother cell as described (Weinert and Hartwell 1988; Lewis *et al.* 1998). Four replicates including 400 cells of each strain were tested.

Southern blot experiments were performed using chemiluminescence and a DIG-labeled telomere DNA probe essentially as described (Becerra *et al.* 2012) except that images were captured using a ChemiDoc imaging instrument (BioRad). Briefly, wild-type cells containing pRS426 and *yku70* cells containing pRS426, pVL715, or pVL735 were shifted from 30°C cultures into 100 ml synthetic glucose minus uracil broth at 3 × 10^6^ cells per ml and shaken overnight at 37°C. Chromosomal DNA was extracted, digested with XhoI, run on a 1.2% agarose gel along with DIG-labeled DNA Molecular Weight Marker III (Roche), transferred to a Hybond-N+ membrane, and probed with a DIG-labeled telomere repeat fragment derived from the plasmid YTCA-1. Telomere sizes were calculated by determining the distances traveled by the six lowest DIG standard bands, plotting their sizes *vs* distances traveled, and determining the equation for the best-fit trendline through all data points. This equation was then used to convert the distance traveled for the lowest band in each sample lane to DNA size in base-pairs.

### Dilution pronging cell survival assays

Cells were harvested into sterile deionized H_2_O from freshly grown patches on plates, diluted, and sonicated for 10 s using a Sonics Vibracell Ultrasonic Processor, quantitated by counting with a hemocytometer, and then 1 × 10^7^, 2 × 10^7^, or 4 × 10^7^ cells (depending on the experiment) were added to water in a final volume of 220 μl in a 96-well microtiter plate. The cells were serially diluted fivefold, 6 times across the length of the dish, and pronged onto either selective synthetic or YPDA plates, depending on the assay. Pronged cells were incubated for 3–4 days at 30°C, 37°C, or 39°C depending upon the assay being performed. Because Petri dish incubators often exhibit temperature gradients, *i.e.*, between the bottom and top sections of the inner chamber, thermometers were placed in covered beakers of water at each level that was used and carefully monitored.

## Results

### Temperature sensitivity of *yku* mutants is rescued by overexpression of telomerase subunits Est2 and *TLC1* RNA, but not Est1 or Est3

Overexpression of either the reverse transcriptase gene or the RNA subunit gene of telomerase suppresses the killing of *yku* cells grown at 37°C (Nugent *et al.* 1998; Teo and Jackson 2001; Lewis *et al.* 2002; Wasko *et al.* 2009; Tong *et al.* 2011). The rescue mechanism is unknown but must involve a process that is able to counter the progressive degradation of telomeres that occurs at high temperature.

**Figure 1:**
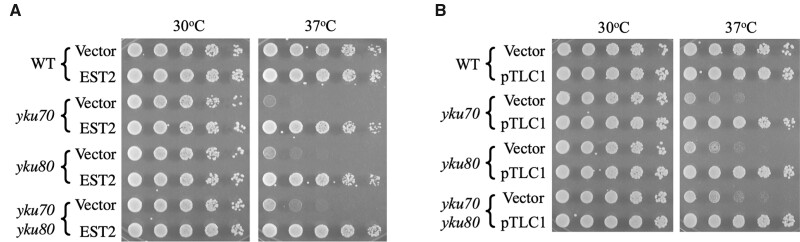
Overexpression of Est2 or *TLC1* RNA suppresses the growth defects of *yku* single and double mutants. Equivalent numbers of cells were harvested and serially diluted fivefold. (A) Plasmid-based overexpression of Est2 (pVL999). (B) Overexpression of *TLC1* RNA (pLKL64Y).

Yku70 and Yku80 normally function together as a heterodimeric DNA end-binding complex, but past work has indicated that each protein can bind with high affinity to several other proteins. Initial tests were performed to determine if rescue by telomerase was equally efficient in cells lacking only Yku70 or only Yku80 and in cells lacking both protein subunits, *i.e.*, *yku70 yku80* double mutants. For these experiments, Est2 and *TLC1* RNA were overexpressed from the yeast *ADH1* promoter on multicopy 2-micron plasmids (pVL999 and pLKL64Y, respectively; see *Materials and Methods*). Briefly, cells with either a control vector or telomerase gene plasmid containing *LEU2* were propagated on synthetic glucose plates lacking leucine (Glu minus Leu) at 30°C, harvested, counted and diluted serially in fivefold increments, and pronged onto Glu minus Leu plates that were subsequently grown at either 30°C or 37°C (Figure  1, A and B). Growth of *yku70* and *yku80* single mutants was rescued by both *EST2* and *TLC1* and the colony numbers and sizes (reflecting survival and growth rates, respectively) were similar to those seen with wild-type cells grown at 37°C. Thus, the fraction of cells overexpressing the subunits that formed colonies (their plating efficiency) was similar to that of wild-type cells. Rescue of mutants lacking both subunits (*yku70 yku80* cells) was as efficient as that seen in the single mutants (Figure  1, A and B).

**Figure 2:**
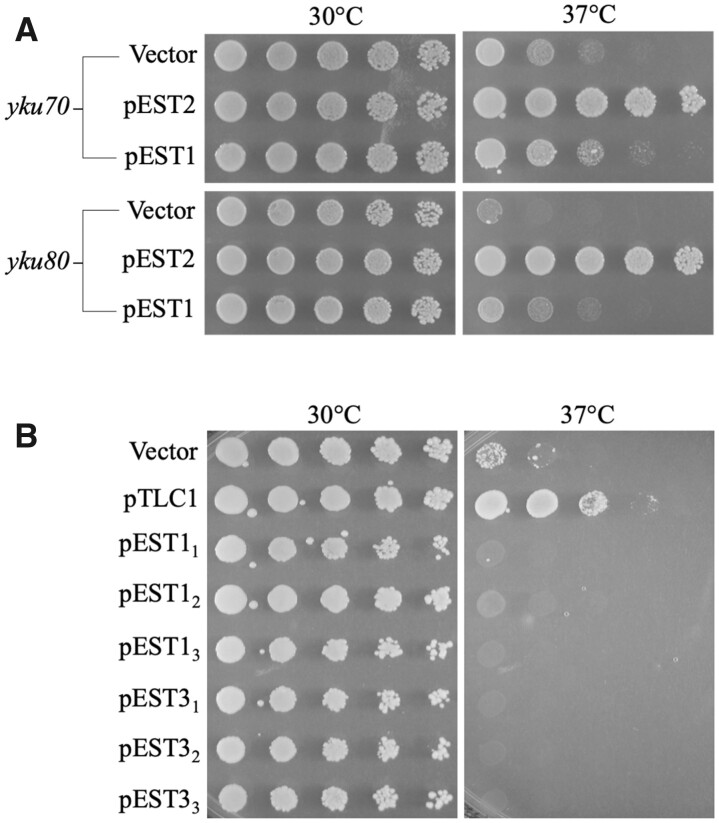
Survival of *yku70* cells at 37°C is not enhanced by overexpression of telomerase subunits Est1 or Est3. (A) High-level expression of Est2 and Est1 using plasmids pVL999 and pVL784. (B) Overexpression of *TLC1* RNA using plasmids pLKL83Y (pTLC1), pLKL92Y (pEST1), and pLKL93Y (pEST3). Three independent isolates of the latter plasmid transformants are shown. Fivefold dilutions were pronged to plates as in Figure  1.

The next experiments were designed to test overexpression of the other core telomerase subunit genes *EST1* and *EST3*. Using previously described plasmids pVL999 (*2µ EST2*) and pVL784 (*2µ EST1*), we observed strong rescue of *yku70* and *yku80* mutants by *EST2*, but not by *EST1* (Figure  2A). A previous report had suggested that *EST1* overexpression could suppress temperature sensitivity in *yku* cells, but another study saw little impact (Nugent *et al.* 1998; Tong *et al.* 2011). For clarification, we cloned, sequenced, and expressed both *EST1* and *EST3* from the strong *S. cerevisiae TEF1* promoter on *2µ* plasmids (pLKL92Y and pLKL93Y, respectively). Three independent isolates of each plasmid were transformed into *yku70* cells and tested for survival. Neither *EST1* nor *EST3* was able to rescue the mutants (Figure  2B). The result with *EST1* is consistent with the results reported in Figure  4B of Tong *et al.* which showed that *EST1* overexpression increased colony sizes slightly but had little impact on colony numbers (survival) in pronging assays. Importantly, results observed in the current study and in that of Tong *et al.* were distinctly different from the orders of magnitude increase in survival caused by Est2 protein or *TLC1* RNA overexpression (Figure  1).

**Figure 3:**
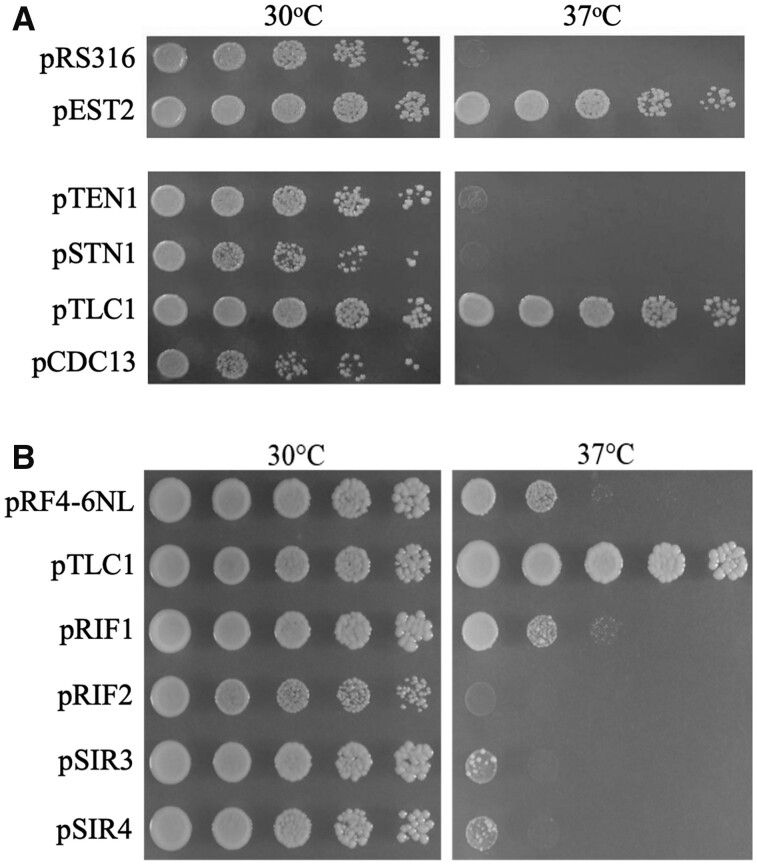
*yku70* inviability is suppressed by overexpression of Est2 and *TLC1* RNA but not other telomerase-associated and telomere-associated proteins. (A) Survival assays employing plasmids pVL715 (pEST2), YEp195-TEN1-HA-HIS (pTEN1), YEP195-STN1-HA-HIS (pSTN1), pLKL83Y (pTLC1), and pVL459 (pCDC13). (B) Survival assays using plasmids pLKL84Y (pTLC1), pRF4-6NL+RIF1 (pRIF1), pRF4-6NL+RIF2 (pRIF2), pRF4-6NL+SIR3 (pSIR3), and pRF4-6NL+SIR4 (pSIR4).

Several proteins are known to interact with telomeres and/or telomerase and therefore might also impact telomere attrition in *yku* mutants at 37°C. Using previously described overexpression plasmids (Chandra *et al.* 2001; Grandin *et al.* 2001), Cdc13 and the Cdc13-binding proteins Stn1 and Ten1 were overexpressed in *yku70* cells. None of the telomerase-interacting proteins could suppress killing at 37°C (Figure  3A). Overexpression of the telomerase regulatory proteins Rif1 and Rif2, as well as the telomere and subtelomere-associated proteins Sir3 and Sir4, were performed next. Elevated levels of these proteins did not suppress the ts^-^ phenotype of *yku70* cells (Figure  3B). Thus, these data indicate that supraphysiological levels of Est2 reverse transcriptase protein and *TLC1* template RNA, but not the other telomerase subunits or telomerase-associated proteins, alleviates the telomere instability and cell death seen in *yku* mutants at elevated temperatures.

### Dependence of rescue by Est2 on the presence of *TLC1* RNA

**Figure 4:**
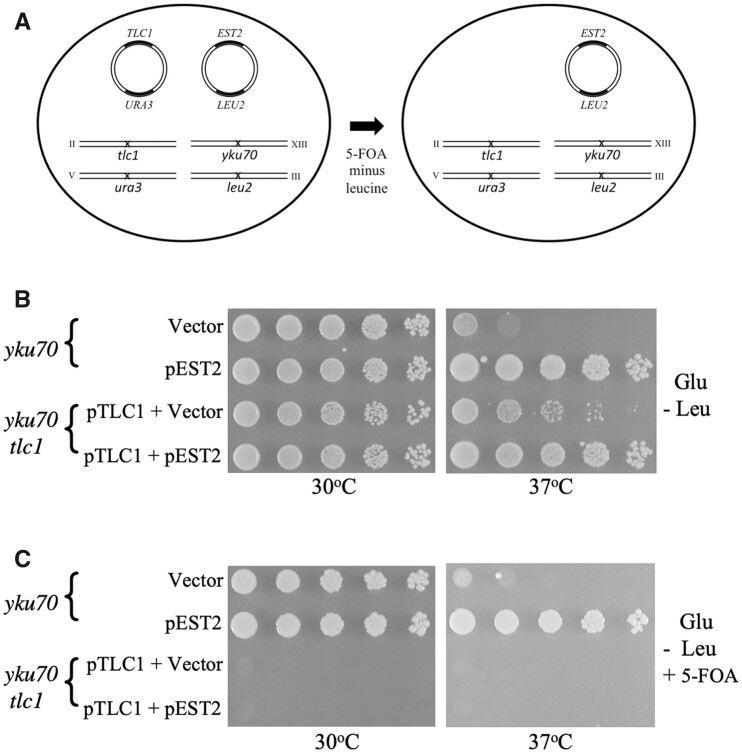
Assessment of the ability of *EST2* to rescue *yku70* mutants in cells lacking another component of the telomerase complex, *TLC1* RNA. (A) Diagrammatic representation of the strain YLKL991 containing the plasmids pLKL83Y (*URA3 ADH1p::TLC1*) and pVL999 (*LEU2 ADH1p::EST2*) before and after transfer to 5-FOA plates. Roman numerals indicated chromosome numbers. (B and C) Growth of *yku70* control mutants and YLKL991 cells (*yku70 tlc1*) containing either the vector pRS425 or the *EST2* plasmid pVL999 at 30°C and 37°C. Glu-Leu, synthetic glucose plates without leucine.

An active telomerase core complex requires Est2 reverse transcriptase, the Est1 and Est3 proteins, and *TLC1* RNA. As an initial test to determine if rescue by Est2 is dependent upon a functional, replication-competent multisubunit complex, the requirement for *TLC1* template RNA was assessed. Strain YLKL991 has *YKU70* and *TLC1* inactivated, but its telomeres are stable because it also contains the 2-micron plasmid pLKL83Y (*ADH1p::TLC1*) (Figure  4A). The *URA3* marker makes it possible to use 5-FOA plates to select for cells that have lost the *TLC1* plasmid, effectively switching cells from a genotype of *yku70* to *yku70 tlc1*. YLKL991 cells were separately transformed with control vector pRS425 (*LEU2*) or pVL999 (*LEU2 ADH1p::EST2*) and propagated on media without leucine and uracil to maintain selection for both plasmids. Transfer of cultures to media containing 0.1% 5-FOA without leucine selects for cells that are overexpressing Est2 in *yku70* mutants that also do not produce *TLC1* RNA (shown schematically in Figure  4A).

The consequences of pronging cells onto plates without leucine and without 5-FOA at 30°C and 37°C are shown in Figure  4B. An isogenic *yku70* strain (YLKL652), also containing either pRS425 or the *EST2* overexpression plasmid pVL999, was included as a control. As expected, cells overexpressing Est2 protein alone or both Est2 and *TLC1* RNA grew robustly at 37°C (Figure  4B). Cells overexpressing the template RNA alone were also rescued, though colony numbers were reduced relative to cells overexpressing both telomerase genes (Figure  4B, 3rd row *vs* 4th row). Pronging of cells onto plates containing 5-FOA revealed that overexpression of Est2 protein did not rescue the growth of *yku70 tlc1* cells at 37°C and also not at 30°C (Figure  4C, 3rd and 4th rows). *tlc1 yku* double mutants grow poorly at 30°C (Nugent *et al.* 1998; Ribes-Zamora *et al.* 2007; Wellinger and Zakian 2012; Teixeira 2013). The results in Figure  4C indicate that the *tlc1 yku70* strains senesced much more rapidly than *tlc1* single mutants at 30°C (over less than the approximately 20 generations needed to form a visible colony) and that neither this phenomenon nor the growth defects at 37°C could be alleviated by the presence of supraphysiological levels of Est2 protein.

### Rescue of *yku70* cells does not require the reverse transcriptase function of telomerase

Two likely mechanisms by which overexpression of the Est2 reverse transcriptase might alleviate telomere shortening and rescue the growth of *yku* mutants at high temperatures are the following: first, higher levels of reverse transcriptase activity may extend the DNA at chromosome ends far enough during each S phase to counteract the losses resulting from increased degradation; alternatively, Est2 protein may bind to telomere DNA and/or a telomere-associated protein and physically block deleterious processing of the ends by Exo1 and possibly other enzymes, *i.e.*, the mechanism may primarily involve structural reinforcement of the cap complex. It is also possible that both mechanisms are involved.

**Figure 5:**
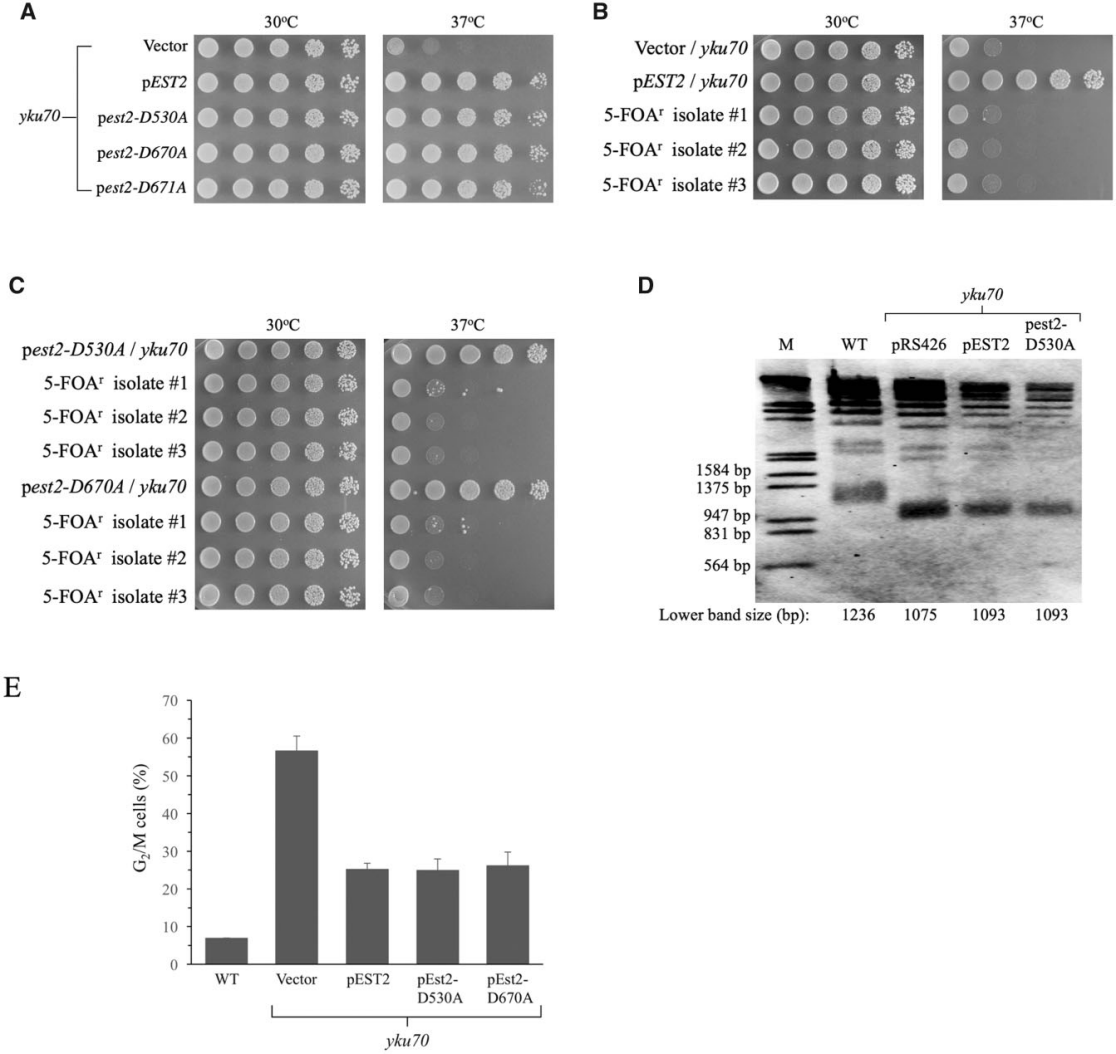
Overexpression of catalytically inactive Est2 reverse transcriptase proteins suppresses killing of *yku70* mutants at high temperatures. (A) Pronging assays were performed using YLKL652 cells containing the control *2µ* vector pRS426 or *2µ* plasmids expressing wild-type or mutant *EST2* genes from an *ADH1* promoter. (B and C) Growth of plasmid-containing and 5FOA^r^ (plasmidless) derivatives of the high temperature-resistant *yku70* cells was assessed. Three independent 5-FOA^r^ isolates of cells that had overexpressed *EST2*, *est2-D530A*, or *est2-D670A* were tested. (D) Overexpression of wild type and inactive Est2 proteins does not alter the short telomere phenotype of *yku70* cells. Cells were propagated at 37°C in glucose broth without uracil, DNA was extracted and digested with XhoI, and the resulting fragments were analyzed by Southern blot. A telomere repeat DNA fragment was employed as probe. M, molecular weight standard. (E) The DNA damage response of *yku70* cells at 37°C is suppressed equally by overexpression of WT Est2 and the enzymatically inactive derivatives Est2-D530A and Est2-D670A. Cells were inoculated into YPDA broth at the same starting titer, shaken for 12 h at 37°C and analyzed by phase-contrast microscopy.

The Cech and Lundblad labs have previously developed and characterized several *EST2* gene mutants (Lingner *et al.* 1997). Three of the mutants (*est2-D530A*, *est2-D670A*, and *est2-D671A*) produce proteins that are stable *in vivo* but catalytically inactive. est2-Δ cells expressing the mutant proteins from plasmids exhibited senescence (loss of growth capability after approximately 70 generations). Expression of the mutant proteins in wild-type (*EST2*) strains produced dominant negative inhibition of telomerase activity and shorter telomeres, suggesting that the mutant proteins could form complexes with other subunits but that these complexes did not retain polymerase activity. Furthermore, two of the mutant proteins (Est2-D530A and Est2-D671A) lacked detectable telomerase activity when protein extracts were prepared and tested (Lingner *et al.* 1997). Plasmids expressing either wild-type *EST2* or the three mutant genes were transformed into the *yku70* strain YLKL652 and tested for growth at 30°C and 37°C. Strikingly, overexpression of each of the reverse transcriptase-defective proteins could rescue the inviability of *yku70* cells as efficiently as overexpression of the wild-type Est2 protein (Figure  5A, compare rows 3–5 *vs* row 2 at 37°C).

Although high-level expression of the mutant proteins leads to the formation of inactive Est2^m^-Est1-Est3-TLC1 RNA complexes inside cells, no negative effects on cell growth were observed in the BY4742 strains employed for these studies and overexpression did not lead to telomere shortening-induced senescence (Supplementary Figure S1). A possible explanation for the reduced impact of the mutant Est2 proteins on cell growth relative to results seen in the earlier study by Lingner *et al.* (1997) may be differences in plasmid copy number, which can vary among yeast strains (Futcher and Cox 1984; Flagg *et al.* 2019). To monitor senescence, wild-type Est2 and two mutant Est2 proteins were separately overexpressed in normal cells and in *yku70* mutants. Cells were repeatedly picked and streaked from small colonies on one plate to a new plate, allowing growth at 30°C for 3–4 days after each streak as previously described (Becerra *et al.* 2012; Ballew and Lundblad 2013). Control telomerase-deficient *est2* cells exhibited senescence, or loss of growth capability due to extreme telomere shortening, on the 5th streak plate under these conditions, corresponding to growth for approximately 70 generations, calculated as previously described (Becerra *et al.* 2012; Supplementary Figure S1A). Overexpression of the wild-type Est2, Est2-D530A, or Est2-D670A proteins did not cause detectable senescence after up to ∼130 generations of growth, (Supplementary Figure S1, B and C).

To confirm that rescue of the *yku70* cells by the mutant proteins shown in Figure  5A was dependent on synthesis of the plasmid-encoded proteins (ruling out possible genetic or epigenetic suppressor effects in the *yku70* strains or other unlikely mechanisms), rescued cells containing pVL999 (*EST2*), pVL735 (*est2-D530A*) or pVL743 (*est2-D670A*) were streaked to 5-FOA plates to select for cells that had lost the rescuing plasmids. Three independent 5-FOA^r^ colonies were then tested for survival at 37°C. Loss of the telomerase gene on the plasmid led to loss of the rescue phenotype at 37°C for each of the alleles (Figure  5, B and C). These results demonstrate that suppression required continuous overexpression of either wild type or catalytically inactive Est2 proteins from the plasmids.

In additional tests, the impact of overexpressing the wild type and mutant telomerase proteins on telomere lengths was assessed. Chromosomal DNAs were purified, digested with XhoI, and probed with a digoxigenin-labeled DNA probe as previously described (Becerra *et al.* 2012). As shown in the Southern blot in Figure  5D, the short telomeres seen in *yku70* strains were not lengthened appreciably by overexpression of either wild-type Est2 or Est2-D530A protein (this was also true for Est2-D670A; not shown). Quantification of telomere shortening is done by measuring the size of the lowest telomere band after digestion with XhoI (Becerra *et al.* 2012). Graphical analysis comparing molecular weight standard and sample bands revealed that the lowest XhoI-generated telomere band in wild-type cells was 1236 bp in size. By contrast, the equivalent fragments were smaller, ranging from 1075 to 1093 bp, in the *yku70* strains (Figure  5D). Thus, overexpression of the wild-type and mutant Est2 proteins did not have a large impact on the lengths of the short telomeres in the *yku70* mutants.

It is well-established that growth of *yku70* cells at elevated temperatures leads to strong accumulation of large-budded G_2_ phase cells due to telomere degradation-induced activation of the DNA damage checkpoint response (Teo and Jackson 2001; Maringele and Lydall 2002; Fisher and Zakian 2005; Clerici *et al.* 2008; Becerra *et al.* 2012; Fell and Schild-Poulter 2015). When *yku70* cells grown at 30°C in YPDA broth were shifted to 37°C, the fraction of arrested large-budded cells increased to become almost 60% of all cells within 12 h (Figure  5E, vector control). This phenotype was strongly suppressed by overexpression of the Est2, Est2-D530A, and Est2-D670A proteins and the level of suppression was similar among each of the Est2 proteins. The comparable effects observed with both catalytically active and inactive Est2 proteins are in accord with the identical survival outcomes presented in Figure  5A.

### Impact of overexpression of truncated *TLC1* RNAs on survival of *yku* mutants

**Figure 6:**
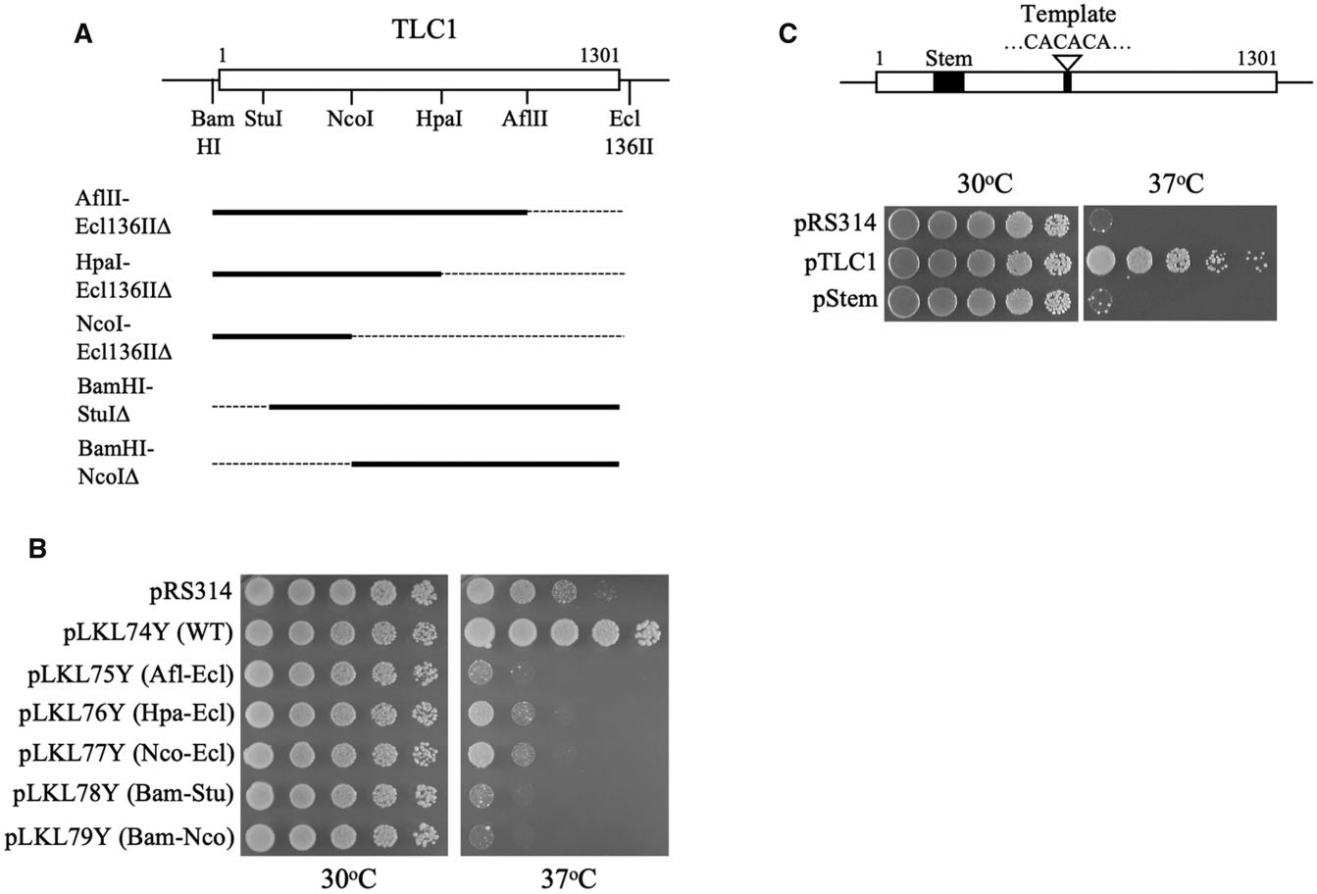
The inviability of *yku70* cells is not suppressed by truncated *TLC1* RNAs. (A) Schematic representation of *TLC1* gene and the deleted segments (thin lines). (B) Survival of *yku70* mutants was rescued by overexpression of the full-length RNA, but not the truncated RNAs. (C) Overexpression of the 48 nt Stem region of *TLC1* RNA did not suppress the ts^−^ phenotype of *yku70* cells. pTLC1, plasmid pTRP61; pStem, plasmid pTCG-3xStem.

Overexpression of *TLC1* RNA has been shown to suppress the chemical, radiation, and EcoRI nuclease expression sensitivities of *mre11*, *rad50*, and *xrs2* mutants (Lewis *et al.* 2002). Suppression of DSB repair deficiency was found to require overexpression of only a small region at the 5′ end of the RNA referred to as the Stem region (Peterson *et al.* 2001; Stellwagen *et al*. 2003; Wasko *et al.* 2009). Several deletion derivatives of *TLC1* were overexpressed in *yku70* cells but, in contrast to results with *mrx* mutants, none were able to rescue cell viability at 37°C (Figure  6, A and B). Similarly, overexpression of the RNA stem region that could rescue *mrx* mutants also did not restore growth at 37°C (Figure  6C).

### Role of DSB repair pathway proteins in telomere stabilization by *EST2* or *TLC1*

**Figure 7:**
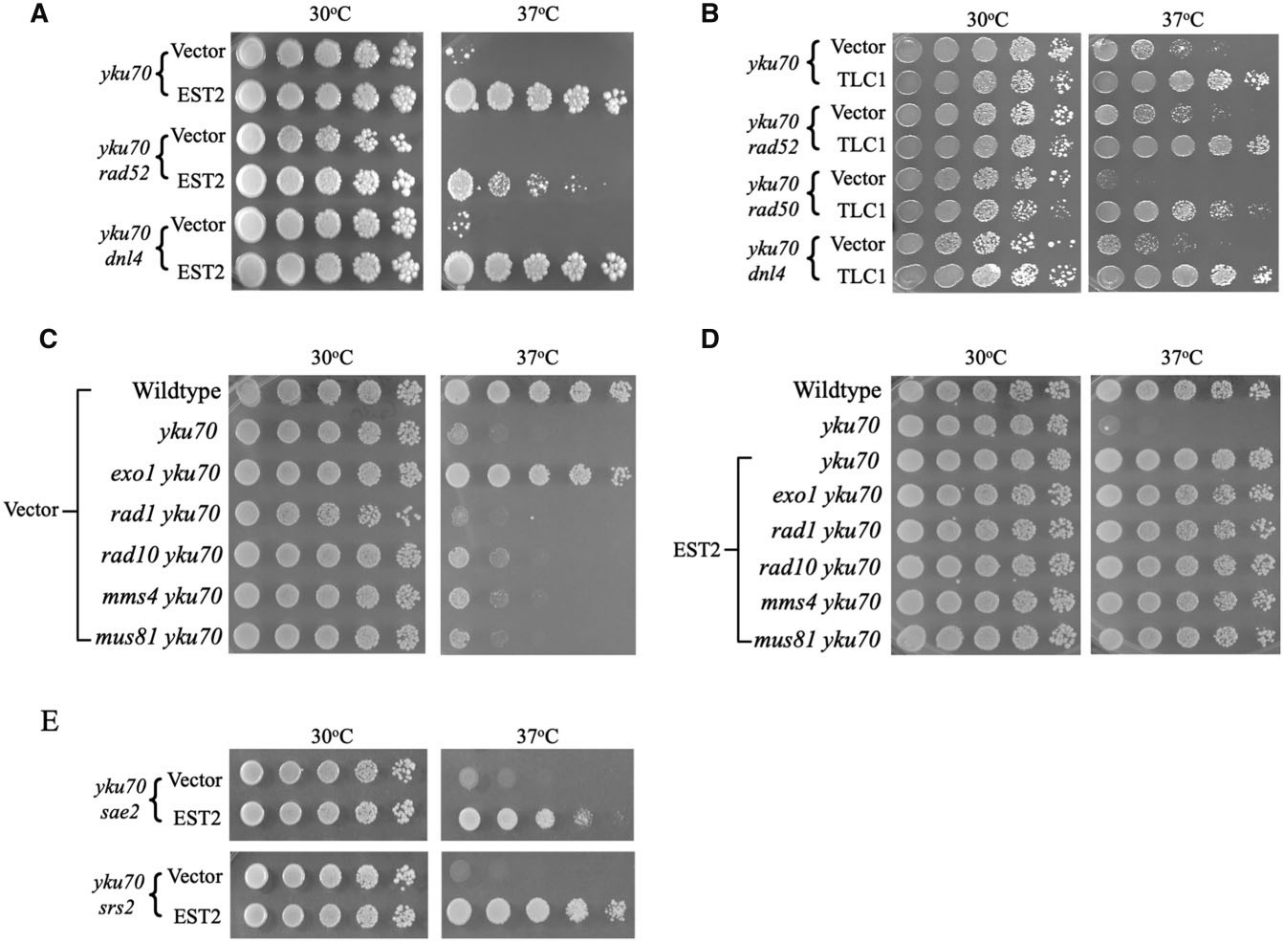
DSB repair pathways regulate telomere length maintenance but are not required for telomere stabilization by Est2 or TLC1 RNA in *yku70* mutants. (A and B) Suppression by *EST2* and *TLC1* is not dependent on HR (*rad52*) or NHEJ (*dnl4*) pathway proteins. (C) Elimination of the Exo1 exonuclease, but not the Rad1/Rad10 or Mms4/Mus81 nucleases, rescues growth of *yku70* mutants. (D) Suppression of lethality by overexpression of Est2 protein is unaffected by inactivation of nuclease genes. (E) Absence of nuclease-stimulatory proteins Sae2 and Srs2 does not affect survival or suppression of *yku70* cells.

The results in Figure  5 demonstrated that Est2 overexpression rescues the telomere capping defect of *yku* mutants by a replication-independent mechanism, likely involving structural reinforcement of the cap. Telomere replication and stability are regulated by proteins acting in multiple pathways, including the two major pathways of DSB repair. New experiments were performed to determine if members of these and other pathways might play a vital role in telomerase-mediated rescue of *yku* cells. To test other components of NHEJ, rescue of *yku70* cells that also had DNA Ligase IV (*DNL4*) inactivated was assessed and found to be similar to that of *yku70* single mutants (Figure  7, A and B). *RAD52* is critical for the repair of DSBs by the HR pathway and is known to affect telomere shortening rates in cells undergoing senescence (Lundblad and Blackburn 1993; Le *et al.* 1999; Gatbonton *et al.* 2006; Gao *et al.* 2010; Becerra *et al.* 2012; Teixeira 2013; Ghanem *et al.* 2019). Growth of *yku70 rad52* double mutants was strongly enhanced at 37°C when either Est2 or *TLC1* RNA was overexpressed, though colony growth was modestly reduced compared to rescued *yku70* single mutants (Figure  7, A and B).

Past studies have demonstrated that inactivation of *EXO1*, encoding a 5′-to-3′ exonuclease active in repair by HR, suppresses the loss of viability in *yku70* strains at 37°C (Maringele and Lydall 2002, 2004; Wellinger and Zakian 2012). In accord with this, we observed strong suppression of the temperature sensitivity phenotype in *yku70 exo1* double mutants (Figure  7C, 3rd row). The potential roles of other nucleases were also analyzed. Inactivation of the Rad1/Rad10 nuclease complex is known to impair processing of broken DNA ends and enhance mutagenesis associated with degraded telomeres during cell senescence (Hackett and Greider 2003; Zhu *et al.* 2003; Mazón *et al.* 2012). The Mms4/Mus81 complex is a structure-specific DNA endonuclease with roles in replication and recombination during mitosis and meiosis (Hickson and Mankouri 2011; Gallo-Fernández *et al.* 2012; Mazón and Symington 2013). Combining null alleles of *rad1*, *rad10*, *mms4*, or *mus81* with *yku70* did not alleviate the temperature sensitivity of these strains (Figure  7C). Furthermore, the ability of Est2 to rescue the inviability of the double mutants at 37°C was similar to that seen in *yku70* single mutants (Figure  7D). The results indicate that, in contrast to Exo1, these nucleases do not play critical roles in telomeric DNA degradation in the absence of Yku and are also not needed for stabilization by overexpression of telomerase.

We also tested the requirement for two additional genes affecting DSB repair: *SAE2*, encoding a protein that stimulates Mrx nuclease activity and plays a role in telomere elongation, and *SRS2*, which encodes a DNA helicase that stimulates the Mus81/Mms4 nuclease and affects telomere shortening and end-to-end fusion formation (Wang and Baumann 2008; Bonetti *et al.* 2009; Hardy *et al.* 2014). We observed that both *yku70 sae2* and *yku70 srs2* mutants were inviable at 37°C (Figure  7E). In addition, suppression by Est2 was robust in the double mutants, indicating that they were not required.

### Requirements for telomeric chromatin-associated proteins in Est2-mediated rescue of *yku70* cells at 37°C

**Figure 8:**
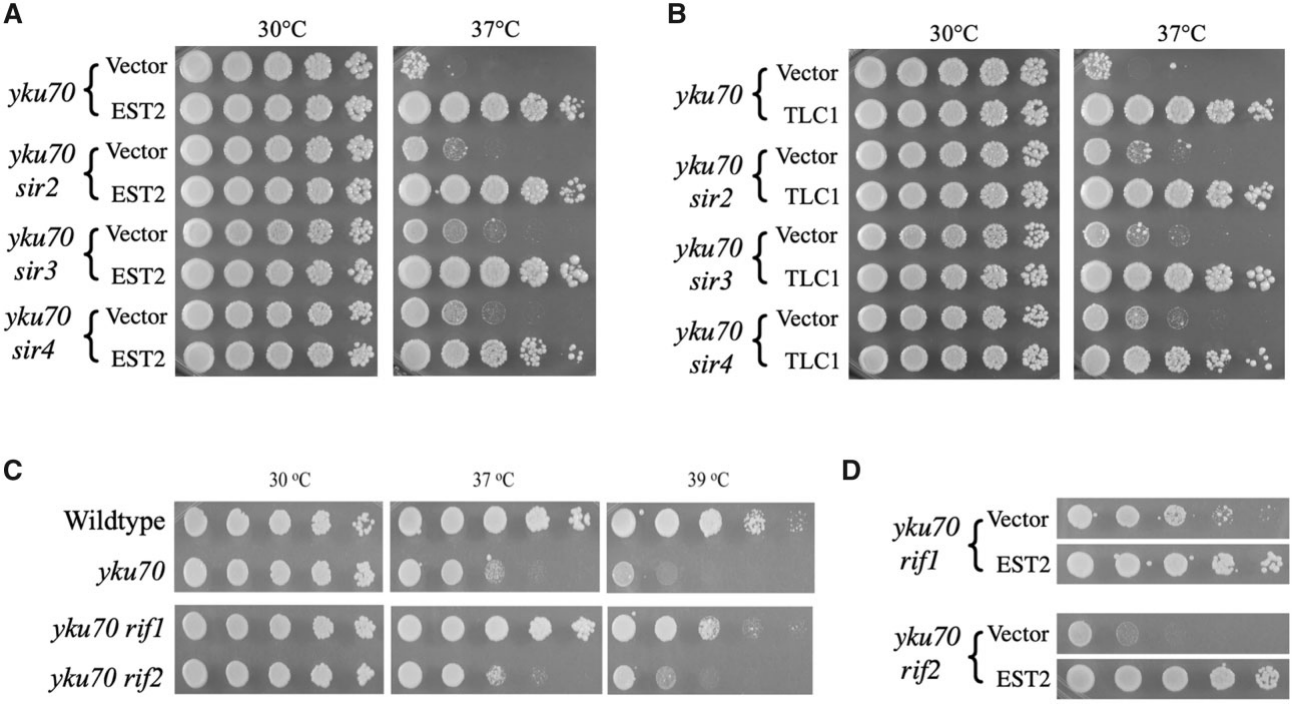
Survival of *yku70* cells is independent of the Sir and Rif proteins. (A and B) Overexpression of Est2 and *TLC1* RNA rescues growth of *yku70 sir* double mutants. (C) The ts^−^ phenotype of *yku70* cells is completely suppressed by co-inactivation of *RIF1* at 37°C but only partially suppressed at 39°C. (D) Overexpression of Est2 rescues growth of *yku70 rif1* and *yku70 rif2* cells at 39°C. The higher temperature of 39°C was employed to accommodate the enhanced resistance of *yku70 rif1* strains.

The Ku and Sirtuin (Sir2, Sir3, and Sir4) complexes mediate the tethering of telomeric DNA ends to the nuclear periphery (Taddei and Gasser 2012; Agmon *et al.* 2013; Avşaroğlu *et al.* 2014). Interestingly, coinactivation of *SIR2*, *SIR3*, or *SIR4* produced a slight increase in growth of *yku70* cells at 37°C, but did not hinder the ability of Est2 or *TLC1* RNA to rescue the cells at high temperature (Figure  8, A and B). The Rif1 and Rif2 proteins regulate the extent of polymerization by Est2 during S phase and maintain transcriptional silencing in telomeric regions. Elimination of negative regulation of telomerase by deletion of *RIF1* (but not *RIF2*) leads to elongated telomeres and has been shown previously to suppress the killing of *yku70* cells at 37°C (Teng *et al.* 2000; Maringele and Lydall 2002; Wellinger and Zakian 2012). Consistent with these past studies, we observed suppression in *yku70 rif1* mutants, but not in *yku70 rif2* cells, at 37°C (Figure  8C, lower panel). Modest cell killing could be detected in the *yku70 rif1* double mutants only when they were grown at the more stringent temperature of 39°C (Figure  8C, right panel). To assess whether Rif1 or Rif2 was required for Est2-mediated suppression, growth of each double mutant was analyzed at 39°C. Survival was restored to wild-type levels in both *yku70 rif1* and *yku70 rif2* cells when Est2 was overexpressed (Figure  8D), indicating that neither protein was essential for suppression.

## Discussion

In this study, we have investigated the mechanism by which overexpression of telomerase can suppress the telomere capping defects and loss of viability of *yku* mutants at elevated temperatures. Initial experiments established that overexpression of Est2 protein or *TLC1* RNA rescued growth of both *yku70* and *yku80* mutants, and *yku70 yku80* double mutants as well. The similarity of phenotypes in the single and double mutants suggests that one subunit did not retain partial function in the absence of the other. It is important to note that the number of colonies formed when Est2 or *TLC1* RNA was overexpressed in *yku* cells was high, usually similar to results obtained when equivalent numbers of wild type or *yku70* cells were simply diluted and pronged onto plates and grown at 30°C. That is, plating efficiencies at both 30°C and 37°C were typically close to 100% and nearly identical for wild-type cells and for mutant cells overexpressing Est2 and *TLC1* RNA. This finding is important because it means that *yku70* suppression does not involve the induction of rare “survivor” cells that may have altered telomere repeat structures (Fellerhoff *et al.* 2000; Maringele and Lydall 2004; Grandin and Charbonneau 2009).

Overexpression of the telomerase core subunits Est1 or Est3, as well as the telomerase-associated proteins Cdc13, Stn1, and Ten1, did not suppress the ts^-^ phenotype of *yku70* cells. This was also true for overexpression of the telomerase regulatory proteins Rif1 and Rif2, as well as Sir3 and Sir4, which are telomeric and subtelomeric chromatin proteins that participate in tethering of telomeres to the nuclear periphery.

Multiple approaches were employed to ascertain whether a functional, replication-competent telomerase complex was required for rescue by Est2. The first experiment employed *yku70 tlc1* cells containing the *TLC1* gene on a *URA3* plasmid plus another plasmid capable of overexpressing Est2. Transfer of the cells to media containing 5-FOA allowed selection for Ura^-^ cells that overexpressed Est2 but did not produce *TLC1* RNA and were therefore incapable of forming productive Est1-Est2-Est3-TLC1 complexes. Overexpression of Est2 did not suppress the loss of viability of these cells at 37°C.

The importance of the replicative activity of Est2 was evaluated by testing the impact of overexpressing three mutant proteins lacking detectable reverse transcriptase activity. Each of the mutant proteins (Est2-D530A, Est2-D670A, and Est2-D671A) suppressed the loss of viability of *yku70* cells at 37°C as efficiently as the wild-type protein. Additional tests revealed that overexpression of wild-type Est2 reverse transcriptase and the mutant enzymes produced equivalent results in tests of their impact on survival, telomere lengths and the G_2_ arrest response of *yku70* cells at 37°C (Figure  5, D and E). Although the catalytically inactive proteins are likely to be capable of binding to other telomerase subunits and potentially creating nonfunctional complexes (Lingner *et al.* 1997), they did not induce senescence in the cells, indicating that sufficient numbers of functional telomerase complexes were present to prevent extreme telomere shortening. These findings are consistent with past Southern blotting experiments indicating that overexpression of *EST2* does not strongly increase average telomere lengths in yeast cells (Teo and Jackson 2001; Williams *et al.* 2014) and also with the telomere length analysis in *yku70* cells shown in Figure  5D.

Rescue by *TLC1* was dissected further by assessing the effects of overexpression of several truncated RNAs. Overexpression of either the native RNA or truncated forms that retained a small stem region near the 5′ end of the RNA were previously shown to suppress the DSB repair deficiencies of *mre11*, *rad50*, and *xrs2* mutants (Peterson *et al.* 2001; Lewis *et al.* 2002; Stellwagen *et al.* 2003). In contrast to results seen with *mrx* mutants, none of the truncated *TLC1* RNAs could rescue growth of *yku70* cells at elevated temperatures (Figure  6). Thus, suppression by *TLC1* could not be localized to a specific subregion within the RNA molecule.

The possibility that other nucleases and other proteins known to impact telomere stability might play a role in *yku70* cell survival and/or suppression by telomerase was investigated by overexpressing Est2 in several double mutants. Rescue of *yku70* cells (1) did not require the NHEJ repair pathway, involving the actions of DNA ligase IV, (2) did not require Rad52, an essential protein of the HR pathway, (3) was independent of structural proteins involved in stabilizing and tethering of telomeres to the nuclear periphery (Sir2, Sir3, and Sir4), and (4) was not dependent upon proteins that regulate elongation by Est2 (Rif1 and Rif2). Although inactivation of the gene encoding Exo1, a 5′-to-3′ exonuclease, as expected, alleviated the inviability of *yku70* strains at 37°C, inactivation of other nuclease genes affecting DSB repair (*RAD1*, *RAD10*, *MMS4*, and *MUS81*) had no effect on either survival at high temperatures or rescue by Est2.

Recombination-deficient *yku70 rad52* cells were strongly rescued by Est2 and *TLC1* RNA overexpression, but growth was consistently modestly lower than that seen when *yku70* single mutants were rescued. It is well-established that Rad52-mediated genetic exchange between telomeres can result in the lengthening of short telomeres and therefore can slow the rate of telomere shortening. For example, telomerase-deficient *est2* or *tlc1* mutants experience telomere shortening and undergo replicative senescence when grown in culture, dividing approximately 70 times before growth capability is lost. However, telomerase-deficient cells can only undergo approximately 40 cell divisions when *RAD52* is coinactivated (Lundblad and Blackburn 1993; Khadaroo *et al.* 2009; Becerra *et al.* 2012; Ghanem *et al.* 2019). Our results suggest that a similar process occurs when *yku70* cells undergo telomere shortening at 37°C, *i.e.*, telomeric DNA is progressively degraded by the actions of Exo1 and possibly other enzymes, but the process is slowed somewhat because a fraction of the shortened telomeres are lengthened via exchanges with other telomeres. In this scenario, the ability of Est2 or *TLC1* RNA to rescue *yku70* cells is not actually dependent upon the HR pathway; rather, it is simply less efficient in recombination-deficient *rad52* strains because they cannot replenish shortened telomeres by unequal genetic exchange processes.

Overexpression of enzymatically inactive Est2 proteins produced strong suppression of *yku70* phenotypes using several different assays in this study. The data, therefore, indicate that overexpression does not involve countering the progressive loss of DNA at 37°C by simply extending the telomeres via new DNA synthesis. Instead, the results point to a structural mechanism for rescue by Est2 and *TLC1* RNA, involving strengthening of residual telomere cap structures to reduce degradation by Exo1 and possibly other DNA processing enzymes.

DNA replication by Est2 occurs during S phase, but crosslinking studies have indicated that the protein remains associated with chromosome ends throughout other phases of the cell cycle (Smith *et al.* 2003; Chan and Blackburn 2004). Although this association is decreased in *yku* cells (Fisher *et al.* 2004), overexpression of the protein may provide relatively constant protection from nucleases once a necessary protein: protein stoichiometry has been achieved. The structural rather than replicative mechanism indicated here is conceptually analogous to the one proposed to explain slowing of telomere shortening in senescing human cells by overexpression of the telomere DNA-binding protein TRF2 (Karlseder *et al.* 2002; and see discussion in Chan and Blackburn 2004). A previous study, Teo and Jackson (2001) demonstrated that ssDNA at telomeres was increased in *yku70* cells shifted to 37°C and remained high even when Est2 was overexpressed. Since cells can have very long tracts of telomeric ssDNA and remain viable (*e.g.*, see Hackett *et al.* 2001; Becerra *et al.* 2012), it is possible that Est2 (and *TLC1* RNA) overexpression doesn’t completely abolish resection. Instead, they may reduce the average extent of ssDNA formed at each telomere and/or reduce the level of overall telomere shortening, thereby preventing catastrophic impacts on the cell.

Several past studies, involving both large high-throughput projects as well as smaller scale Yku70/Yku80-specific protein interaction studies, have suggested that Yku70 can physically interact with a number of proteins. These proteins include Yku80, DNA repair-related proteins Nej1 and Mms21, telomere-associated proteins Sir4 and Mlp2, and several histone proteins (Cherry *et al.* 1998). Evidence suggests that the other subunit, Yku80, also associates with several proteins, including Est1, Est2, and *TLC1* RNA, DNA repair proteins Nej1, Dnl4, and Mre11, telomeric chromatin protein Sir4, chromatin remodeling protein Rsc1, and the RPA complex proteins Rfa1 and Rfa2. Like Yku70, Yku80 is also capable of binding to a number of histone proteins (Cherry *et al.* 1998). A caveat to protein: protein interaction studies is that results detected *in vitro* may not necessarily reflect associations occurring *in vivo* (Meysman *et al.* 2017); however, the potential physiological significance of such interactions is strengthened when they are reproduced using multiple independent methods and/or with multiple members of a known complex as is the case here.

The aggregate physical interaction data suggest that Yku70 and Yku80 are components of larger complexes *in vivo*, potentially consisting of histone and other chromatin-associated proteins and, in a portion of the cell cycle, nuclear porins. We suggest that, in the absence of Yku70 or Yku80 at 37°C, these protective complexes are severely compromised but can be buttressed by association with Est2 protein or *TLC1* RNA throughout most of the cell cycle, creating a barrier that restores resistance to nuclease degradation, especially by Exo1. A previous report indicated that Est2 is involved in Ku-mediated tethering of telomeres to the nuclear periphery (Schober *et al.* 2009). It is possible that restoration of this association contributes to the enhanced survival when Est2 protein is synthesized at elevated levels. We also note that the efficiency of *TLC1* RNA localization to the nucleus is reduced in *yku70* cells (Gallardo *et al.* 2008; Pfingsten *et al.* 2012) and restoration of cell viability may involve relocalization effects as well.

The structure of the eukaryotic telomerase holoenzyme is complex. The yeast enzyme is likely to function as a dimeric conglomerate inside cells and, in addition, the template RNA may associate with several diverse proteins (Prescott and Blackburn 1997; Lin *et al.* 2015; Lemieux *et al.* 2016). Thus, it is likely that the *in vivo* stoichiometry of the complex also plays an important role in the generation of nuclease-resistant structures during telomerase-mediated rescue of *yku70* and *yku80* cells.

## Data availability

The data underlying this article are available in the article and in its online supplementary material.

Supplementary material is available at *G3* online.

## Supplementary Material

jkab359_Supplementary_Data
