# Supplementary figures and images for: Suppression of telomere capping defects of Saccharomyces cerevisiae yku70 and yku80 mutants by telomerase
Source: G3 (Bethesda). 2021 Oct 13;11(12):jkab359. doi: 10.1093/g3journal/jkab359 (PMC8664480; doi:10.1093/g3journal/jkab359)

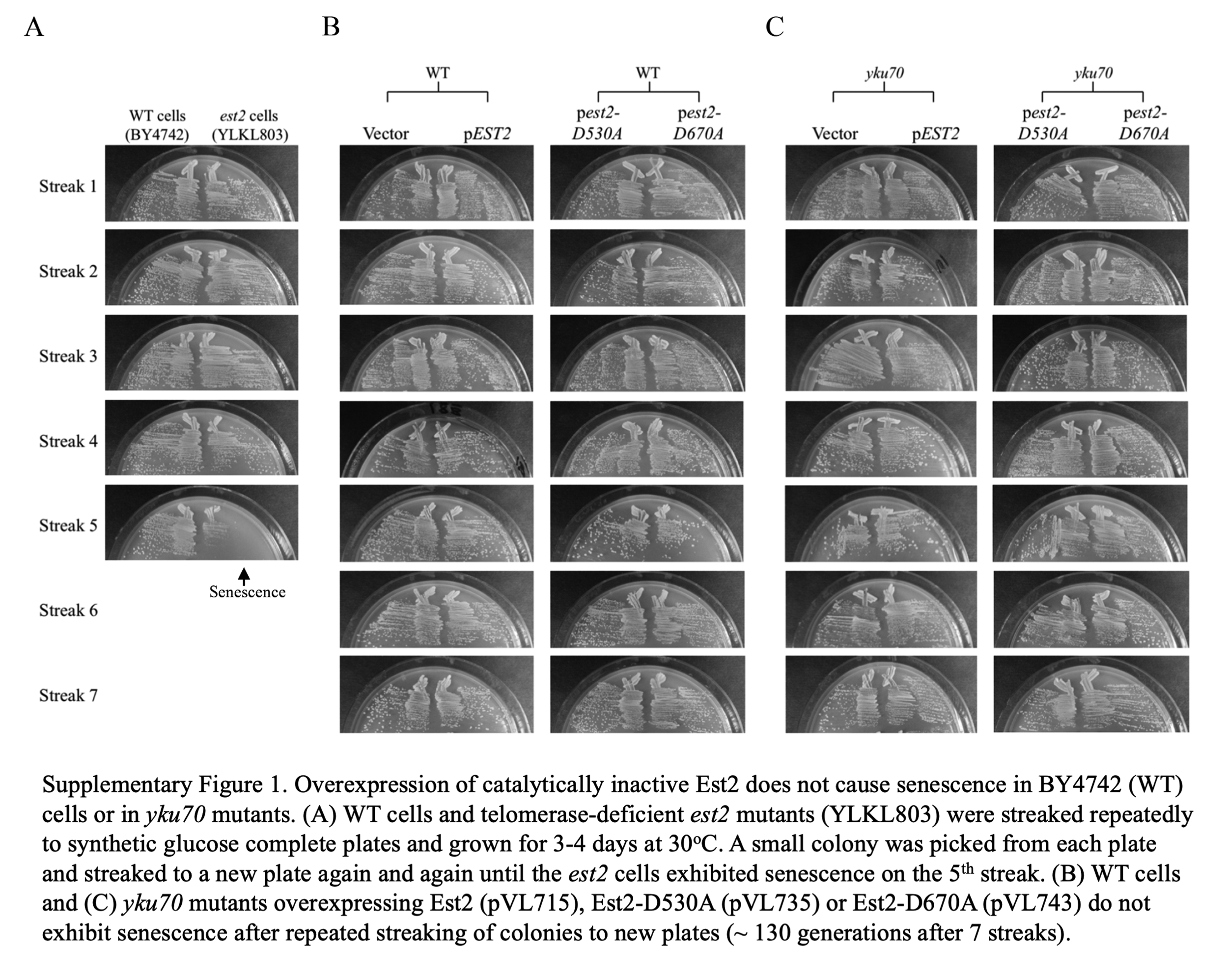

Supplement: jkab359_Supplementary_Data [file jkab359_supplementary_data.zip › jkab359_Supplementary_Data.tif]
